# Occupational burnout in nuclear medicine technologists working in Australia and New Zealand – results of a multi‐national survey

**DOI:** 10.1002/jmrs.834

**Published:** 2024-10-27

**Authors:** Melissa Shields, Daphne James, Lynne McCormack

**Affiliations:** ^1^ The University of Newcastle Callaghan New South Wales Australia

**Keywords:** Professional, psychology, radionuclide imaging (nuclear medicine), research – quantitative

## Abstract

**Introduction:**

Occupational burnout can be associated with negative feelings about the workplace and feeling that a person's efforts are of little consequence. Within a healthcare setting, occupational burnout can be attributed to a high workload or a non‐supportive work environment. Higher levels of burnout are associated with increased absenteeism and turnover, increased medical errors and decreased patient care. The aim of this study was to investigate the levels of occupational burnout within nuclear medicine technologists (NMTs) working in Australia and New Zealand.

**Methods:**

An online questionnaire was distributed via QuestionPro. The questionnaire consisted of four sections, including the Professional Quality of Life Scale (ProQOL) Version 5 (2009) measuring compassion satisfaction, burnout and secondary traumatic stress in the workplace. For this study, only the burnout component of this scale is reported.

**Results:**

There were 162 survey responses. Of the 18 New Zealand participants, 10 (56%) reported moderate levels of burnout. Of the 144 Australian participants, 114 (79%) reported moderate levels of burnout. No NMTs reported high levels of burnout. All states of Australia were represented in the survey, with Queensland, Western Australia and Victoria having the highest number of participants reporting moderate levels of burnout.

**Conclusion:**

This study revealed that more than half of New Zealand participants and three quarters of Australian participants reported moderate levels of burnout. It is imperative to address the wellbeing needs of NMTs working in Australia and New Zealand at an individual and organisational level to support NMTs to be more engaged in their work and help organisations retain staff.

## Graphical abstract

Occupational burnout is associated with negative feelings about the workplace and is often caused by a high workload and a non‐supportive workplace. It is associated with absenteeism, high turnover of staff and decreased patient care. More than half of the New Zealand participants and three quarters of the Australian participants had moderate levels of burnout.

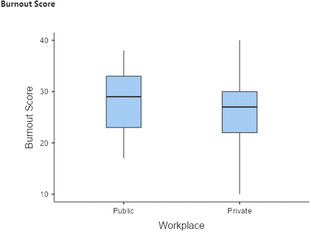

## Introduction

Burnout, classified by the World Health Organization (WHO) in 2020 as an ‘occupational phenomenon’,^1^ was first reported in psychological research by Freudenberger^2^ in the 1970s and has been extensively researched within the healthcare setting since that time.^3^ Occupational burnout is defined by Maslach et al. as three dimensions: emotional exhaustion, depersonalisation and reduced personal accomplishment.^4^, ^5^ Exhaustion presents as chronic feelings of tiredness and fatigue (both mentally and physically)^6^ due to workload.^3^ Depersonalisation, also known as cynicism, is negative feelings and inappropriate attitudes towards work and detachment from work.^3^, ^7^ Within burnout reduced personal accomplishment, or a sense of professional inefficiency,^7^ relates to feelings of reduced capability, sense of incompetence and lack of accomplishment.^7^ Causes of occupational burnout in health professionals include a lack of support and/or respect from management, excessive work hours and workload and a lack of career progression.^8^ Health care workers suffering from occupational burnout have been found to experience a decreased level of empathy, lower productivity and reduced patient care.^9^

An individual's physical and mental health negatively affected by occupational burnout, includes the potential to develop conditions such as anxiety, depression, Type 2 diabetes and heart disease.^10^, ^11^, ^12^ Organisational issues, such as absenteeism, increased turnover of staff and a perceived risk to patient safety^10^, ^11^, ^12^ are often a consequence of occupational burnout. There are many different validated, self‐reporting questionnaires available to measure occupational burnout, including the Maslach Burnout Inventory, Oldenburg Burnout Inventory and Professional Quality of Life Scale.^13^, ^14^ These measuring tools are not recommended as diagnostic tests for occupational burnout, more a guide to raise awareness that a person may be at a higher risk of occupational burnout.^14^, ^15^

In Australia, nuclear medicine technologists (NMTs) complete a four‐year undergraduate degree specifically in nuclear medicine technology. Whereas in New Zealand (NZ), NMTs complete a post‐graduate qualification in nuclear medicine after initially studying medical imaging or a health/science related field. Whilst there is a difference in the education model between the two countries, the scope of practice is the same. The technical responsibilities of NMTs working in Australia and New Zealand are to prepare and administer radiopharmaceuticals to patients, as well as image patients and perform computer analysis on the images.^16^ Excellent communication and patient care skills are also vital skills for NMTs working in Australia and New Zealand. This is due to the need to work closely with patients regularly for extended periods of time where patients require clear instruction prior to and during imaging procedures. NMTs work with a diverse population of patients across the lifespan including those who need varying degrees of medical care, and culturally and linguistically diverse patients. NMTs need to gain the patient's confidence, to ensure patient compliance and comfort throughout the procedure as well as monitor the patient's condition to ensure their safety whilst in the nuclear medicine department.^17^

Within Australia, nuclear medicine departments can be located within public hospitals, funded by state governments or can be stand‐alone private practices, owned by private companies. Geographically, the Australian health care system is grouped into metropolitan, regional, rural and remote areas under the Modified Monash Model (MMM).^18^ This classification is dependent on the road distance to the closest town and population size. Due to their geographical nature, there are no nuclear medicine departments in remote communities, and very few in smaller rural towns. New Zealand's nuclear medicine departments can also be in public hospitals or private departments, although with a different funding scheme. The majority of nuclear medicine departments located in NZ are situated in metropolitan areas, with a very limited number serving the regional and rural communities. In either country, the case load and staffing numbers can vary widely between the public and private system and the geographical location of the practice.

There is a lack of international research into burnout in NMTs,^19^ with only three survey studies^20^, ^21^, ^22^ and one qualitative study^23^ published since 2000. In 2000, more reported moderate levels of occupational burnout overall in NMTs working in Ohio, USA.^20^ As part of a wider study reporting on occupational burnout in radiographers in a large tertiary hospital in Saudi Arabia, Alyousef et al.^21^ found that 42% of participating NMTs had a moderate risk of burnout.^21^ Another study by Shubayr et al.^22^ conducted in 2022 found the subgroup of NMTs working in Saudi Arabia had moderate levels of burnout. In 2023, Shields et al.^23^ conducted a qualitative study utilising Interpretative Phenomenological Analysis (IPA) on a group of NMTs working in a metropolitan area of Australia. This study, exploring the ‘lived’ experiences of a small group of NMTs, concluded that there was an increased risk of burnout amongst the participants. However, as participants matured and gained life experience, they were able to develop their personal resources which helped protect them from this risk of burnout. Being an IPA study, the results could not be generalised to the wider NMT population; however, the findings informed further nomothetic research.

The aim of this study was to investigate the levels of occupational burnout within NMTs working in Australia and New Zealand and to assess the factors that may contribute to workplace stress and potential occupational burnout. This article will focus on the levels of occupational burnout only.

## Methods

University Human Research Ethics Approval was obtained prior to the commencement of the study (H‐2020‐0085). Once developed, the questionnaire was piloted by a small group of NMTs. The final version of the questionnaire (Appendix S1) consisted of closed and open questions divided into four sections; Part A contained demographic and workplace questions; Part B consisted of two self‐assessment questions asking about the perceived stress levels of participants based on a 10‐point Likert scale ranging from 1 (not stressed at all) to 10 (extremely stressed) and the Professional Quality of Life (ProQOL) Version 5 (2009). Part C contained questions pertaining to factors that may contribute to workplace stress and occupational burnout, and Part D comprised of questions regarding methods used by participants to alleviate workplace stress. Only the results from Parts A and B will be discussed in this paper.

The ProQOL (version 5), developed by Stamm over a period from 2009 to 2012, was selected as the most appropriate measurement tool for this study design. Unlike the Maslach Burnout Inventory (MBI), the most used burnout measurement tool, the ProQOL is freely available, the items are slightly adjustable to suit the target audience, and it assesses both positive and negative aspects of professional care. The ProQOL (version 5) is a validated self‐reporting scale that measures compassion satisfaction (CS) and compassion fatigue in the workplace. Compassion fatigue is further broken down into burnout (BO) and secondary traumatic stress (STS). It consists of 30 items (or questions) (10 for each subscale) that ask participants to reflect on how frequently they have experienced a particular feeling/event in the past 30 days against a Likert scale ranging from 1 (never) to 5 (very often) (note there are articles in press using the old (version 4) scoring of 0 (never) to 4 (very often)). Scores are individually summed for CS, BO and STS (with certain scores reversed) and can be compared to a cut‐off scale (Table 1) to illustrate the participant's level (low, moderate or high) of CS, BO or STS. This paper will report on the results from the burnout subscale only. It should be noted that the ProQOL is not a diagnostic tool and should not be used to diagnose any type of mental disorder. It can, however, be used as a screening tool to raise awareness of an individual's balance of positive and negative workplace experiences.^14^

**Table 1:** ProQOL Burnout Scale.^14^

Sum of burnout questions	Level of burnout
22 or less	Low
23–41	Moderate
42 or more	High

The questionnaire was administered online via Questionpro (Survey Analytics LLC, USA) and was open for 6 months from May to November 2023. Participants included any practicing NMT working in Australia or New Zealand. An invitation to participate was first disseminated at the 2023 Australia and New Zealand Society of Nuclear Medicine (ANZSNM) Annual Scientific Conference, and consequently advertised in the ANZSNM Gamma Gazette (2023 Winter Issue). The participant information statement (PIS) and a link to the online questionnaire were promoted on social media accounts of the ANZSNM and its branches, as well as at branch meetings throughout this period. It was also circulated to major private nuclear medicine companies throughout Australia. Due to government health policy, recruitment information was not sent directly to any public hospital nuclear medicine departments. Implied consent was assumed once the participant completed the questionnaire. Participants could withdraw from the questionnaire at any time prior to completing the questionnaire; however, they were unable to withdraw once the questionnaire was complete due to the anonymous nature of the collection method.

Data analysis was performed using descriptive and inferential statistics (independent samples t‐test and one‐way ANOVA) using the Jamovi platform (version 2.3.26). Statistical significance was reached if the test produced a result of less than 0.05.

## Results

### Demographics

Of the approximately 1300 registered NMTs in Australia and New Zealand, 162 completed the questionnaire. It is, however, difficult to determine an accurate response rate as it is unsure how many technologists received an invitation to participate because of the recruitment methods. Of these participants, 124 (76.5%) identified as female, and 144 (88.9%) were from Australia. All states and territories of Australia and New Zealand were represented. Other demographic information can be found in Table 2.

**Table 2:** Participant demographics.

Participant demographics	*n* (%)
Nationality	
Australia	144 (88.9)
New Zealand	18 (11.1)
Gender	
Male	38 (23.5)
Female	124 (76.5)
Years practicing	
0–5	44 (27.2)
6–10	35 (21.6)
11–15	18 (11.1)
16–20	26 (16.0)
21–25	19 (11.7)
26–30	10 (6.2)
30+	10 (6.2)
Employment status	
Full‐time	124 (76.5)
Part‐time	38 (23.5)
Australian states and territories	
New South Wales	31 (21.5)
Queensland	35 (24.3)
Victoria	28 (19.4)
South Australia	21 (14.6)
Western Australia	16 (11.1)
Northern Territory	2 (1.4)
Australian Capital Territory	9 (6.3)
Tasmania	2 (1.4)
Workplace setting	
Public Hospital	75 (46.3)
Private Department	86 (53.1)
Role	
Assistant Technologist	1 (0.6)
Technologist	58 (35.8)
Student Supervisor	1 (0.6)
Research Technologist	1 (0.6)
Senior Technologist	54 (33.3)
Deputy Chief Technologist	14 (8.6)
Chief Technologist	32 (19.8)
Operations Manager	1 (0.6)

### ProQOL

Results from the ProQOL section of the questionnaire found that overall, participants reported a moderate level of burnout (mean = 27.4). There were 124 participants who scored within the moderate level of burnout and 38 who scored within the low level of burnout. There were no participants who scored within the high level of burnout. The highest burnout score was 40 and the lowest was 10. Of the 18 New Zealand participants, 10 (55%) scored within the moderate burnout range (mean = 24.5), and of the 144 Australian participants, 114 (79%) were within the moderate burnout range (mean = 27.78). Other burnout scores relevant to specific participant demographics are in Table 3.

**Table 3:** Burnout scores and statistical results for specific participant demographics.

Participant demographics	Burnout score mean (SD)	*P*‐value	Odds ratio
Nationality			
Australia	27.8 (5.73)		
New Zealand	24.5 (6.06)		
Gender			
Male	27.7 (5.71)	0.727	
Female	27.3 (5.87)		
Years practicing			
0–5	27.6 (5.06)	0.128	
6–10	28.1 (4.81)		
11–15	27.9 (6.00)		
16–20	27.3 (6.72)		
21–25	23.9 (6.98)		
26–30	29.8 (5.18)		
30+	29.1 (6.51)		
Employment status			
Full‐time	28.1 (5.46)	0.010*	1.098 (95% CI 1.028–1.170)
Part‐time	25.1 (6.46)		
Australian States and Territories			
New South Wales	26.4 (5.49)	0.281	
Queensland	29.5 (4.67)		
Victoria	29.0 (5.58)		
South Australia	27.5 (6.05)		
Western Australia	28.6 (6.58)		
Northern Territory	24.0 (7.07)		
Australian Capital Territory	23.0 (5.87)		
Tasmania	25.0 (5.66)		
Workplace setting			
Public Hospital	29.0 (5.66)	0.037*	0.944 (95% CI 0.893–0.997)
Private Department	27.0 (5.87)		
Australian workplace region			
Metropolitan	28.3 (5.57)	0.159	
Regional/Rural	26.8 (5.96)		
Job role			
Chief NMT	27.1 (6.72)	0.373	
Deputy Chief NMT	25.8 (3.51)		
Senior NMT	27.1 (5.97)		
NMT	28.5 (5.69)		

All burnout scores are within the moderate range (22–41).

* *P* < 0.05.

### Statistical analysis

Burnout scores for gender type (male/female), employment status (full‐time/part‐time), workplace type (public/private) and region of employment (metropolitan/regional and rural, for Australian participants only) were compared using an independent samples t‐test (after testing for homogeneity of variance), with the null hypothesis that they were not equal and the significance level set at less than 0.05. Burnout scores for years practicing as a NMT were compared using a one‐way ANOVA (Fisher's) test. This test was also used to compare the burnout scores for NMTs working in Queensland, Victoria and NSW (with these three states having the largest number of participants), as well as to compare the burnout scores for the various job roles held by NMTs working in Australia and New Zealand (Chief NMTs, Deputy Chief NMTs, Senior NMTs and NMTs). There was no statistically significant difference between the burnout scores for males and females, the region of employment (for Australian participants), the three Australian states, the various job roles of NMTs, or for the years of practice for participants. There were, however, statistically significant differences between the workplace type (*P* = 0.037) with an odds ratio of private workplace verse public workplace equalling 0.944 (95% CI 0.893–0.997) and the employment status of participants (*P* = 0.010) with an odds ratio of full‐time employment verse part‐time employment equalling 1.098 (95% CI 1.028–1.170). Statistical results are shown in Table 3.

## Discussion

This is the first cross‐sectional survey study to investigate occupational burnout in NMTs working in Australia and New Zealand. It is difficult to compare these results with other studies, as there is very limited NMT data worldwide, and varying burnout measurement tools have been used. All three previous survey studies^20^, ^21^, ^22^ indicated that the mean scores for participants were in the moderate range for occupational burnout. In this current study, there were no NMTs that scored in the high burnout range, whereas in the previous studies over 25% of participants had scores in the high level of burnout.^20^, ^21^, ^22^ This could be attributed to many different factors, including workplace settings, workload and measurement tool used. All previous survey studies used the Maslach Burnout Inventory (MBI) to measure occupational burnout, where this study used the ProQOL. Both are validated burnout measurement tools^4^, ^14^; however, they use differently worded questions or statements to measure various aspects of occupational burnout. They also score differently and therefore have different values for the different levels of burnout. What is regarded as moderate burnout for one measurement tool could be in the high burnout range for a different measurement tool.^19^ The ProQOL sums the scores for the burnout scale and ranks it against low, moderate or high levels of burnout, whereas the latest version (4^th^ ed. 2016) of the MBI specifies that the rankings of low, moderate and high levels of burnout have been removed as they have no diagnostic validity^14^, ^15^; however, many studies^21^, ^24^, ^25^, ^26^ continue to use this ranking for ease of conveying significance of results.

It should be noted that the three documented studies investigating occupational burnout in NMTs were performed pre‐COVID‐19 pandemic and this study was post‐pandemic; however, the results found very little perceived change in occupational burnout levels. There are a number of studies^22^, ^24^, ^25^ investigating occupational burnout in the medical imaging and radiological science profession during the COVID‐19 pandemic, but only one study by Zanardo et al.^26^ comparing results pre‐ and post‐pandemic. This study reported that radiation therapists working in Italy had very similar (albeit high) levels of burnout pre‐ and post‐COVID‐19 pandemic.^26^ This stable level of reported burnout is in keeping with what has been reported in NMTs, although they were different studies with different demographics using different burnout measurements; hence, it is difficult to make direct comparisons of the results.

In this study, there was no statistical difference between the mean burnout scores for males and females. Although using different burnout measurement tools, this corresponds to other NMT and radiation therapist burnout research that compared burnout scores between genders.^20^, ^27^, ^28^, ^29^ Within the databank of donated ProQOL results from over 1250 cases in multiple international studies across various demographic and workplace settings,^14^ there was no statistical difference between the burnout score means for male and female which also corresponds to the results of this study. These results are supported by Maslach et al.,^11^ who reported that gender is not a strong predictor of burnout in burnout research overall.

Even though the mean burnout score for NMTs working in metropolitan areas within Australia was slightly higher than their regional and rural colleagues, this was not a significant difference. Working in different regions is associated with different workplace stresses,^30^, ^31^ including workload and recruitment/retention of staff, all known contributors to occupational burnout. Whilst there is no data comparing occupational burnout in NMTs or other medical radiation science (MRS) professions working in different regions of Australia, there are data on other healthcare workers. In a 2015 study,^32^ Singh et al. reported that there was no difference in the levels of burnout in mental health nurses working in metropolitan and regional/rural Australia. However, Tham et al.^31^ conducted a survey of almost 8000 Australian healthcare workers in 2020 which determined that rural healthcare workers had higher levels of burnout compared to their metropolitan counterparts. This could have been due to difficulties in recruitment and retention, less availability of locum healthcare workers (for leave relief) and infrastructure challenges.^31^ However, NMTs working regionally and rurally in Australia are also faced with these issues, yet only showed minor differences in burnout scores. This could be because there is an Australia‐wide NMT workforce shortage at present,^33^ affecting both metropolitan and regional/rural workplaces.

This study reports the difference between mean scores of public and private workplaces is significantly significant (*P* = 0.037) and the odds ratio indicates that NMTs in the private sector are less likely than their public hospital colleagues to be at risk of burnout. However, the mean burnout scores were different by two points, and both were well within the moderate level of burnout. Again, there are limited data to compare these results to; however, More (2001) showed no significant difference when comparing NMTs working in hospitals with those not working in hospitals.^20^ A study conducted by Eslick and Raj in 2002,^30^ evaluating workplace stress (a known contributor to occupational burnout) in radiographers working in Sydney, Australia, found no difference in the stress levels between radiographers in the public and private systems. However, the workplace stressors were different between the two workplaces. Radiographers working in private practice found the patients and workload to be their top two stressors, whereas their colleagues employed in the public system listed on‐call/overtime and workload as the top two workplace stressors.^30^ From the results of this study, 60% of NMTs who worked in the public system participated in on‐call, with the majority doing on‐call on a monthly basis and half of those got called in one to two times for the week they were on‐call. Whereas only 19% of the NMTs working in private departments participated in on‐call with over half of these rarely getting called in. Therefore, being called in after‐hours may be a contributing factor to the higher burnout score for NMTs working in the public sector in this study.

In our study, Queensland had the highest burnout score compared to other states of Australia. Queensland is the third largest employer of NMTs in Australia (17% of registered NMTs), behind NSW (35%) and Victoria (27%).^34^ Although it covers a much larger geographical land mass (1.7 million km^2^, compared to NSW 801,000 km^2^ and Victoria 227,000 km^2^) it has a lower population (5.5 million) than the other two states (NSW 8.3 million and Victoria 6.8 million).^35^ Being such a geographically large state, it is challenging to recruit NMTs to the regional areas due to likely limitations in their scope of practice because of limited technologies available,^36^ as well as being potentially long distances away from various support networks (e.g. social). There is no undergraduate nuclear medicine degree within Queensland and therefore, this may also affect recruitment and retention of NMTs, adding to workforce shortages, workplace stress and potential occupational burnout.

This study found that participants working part‐time had a mean burnout score that was significantly lower than their full‐time colleagues (*P* = 0.010), although both part‐time and full‐time NMTs were still in the moderate range of burnout. The odds ratio calculation also revealed that full‐time NMTs were more likely at risk of occupational burnout than those working part‐time. Whilst this corresponds to the findings from the More study in 2001,^20^ there is conflicting data in other health care professions.^27^, ^37^, ^38^, ^39^ Contrary to general presumptions, part‐time employees do not have a lower risk of burnout compared to their full‐time counterparts.^38^ Although they are reducing their workload and, in theory, reducing one of the known causes of burnout, there may be other issues within the workplace that are contributing to the burnout risk, or the number of part‐time hours worked per week is still too high a workload for that individual person. Part‐time employees may have less hours exposed to workplace stressors, but it could be said that part‐time employees have more outside work demands (e.g. family responsibilities).^38^

The literature has reported that burnout levels are higher earlier in a career, when employees are less experienced and do not have the coping mechanisms to manage excessive job demands.^11^, ^32^ This has been found in some MRS professions^39^, ^40^ however, other research has shown no significant differences between years of experience and burnout levels in MRS professionals.^27^, ^29^ The findings from this study showed that participants late in their career (more than 26 years of experience) had higher levels of burnout. This could be due to the increase in personal caregiving demands faced by those late in their career, depleting their personal resources that help counteract job demands. This in turn affects their work engagement and increases their risk of burnout.^41^ Older employers may also have higher levels of burnout as they are less likely to be achieving meaningful goals (or they have already achieved their goals earlier in their career); therefore, they have lower levels of personal accomplishment and a higher risk of burnout.^42^

The results from this survey have suggested a number of reasons why a particular participant group may or may not be at a higher risk of occupational burnout. However, it has been shown that occupational burnout does not follow the exact same pattern in each individual.^3^ Individuals' personalities may affect their interpretations and therefore reactions to their working environment, making them more susceptible to occupational burnout.^10^ An individual's general wellbeing as well as their work‐related wellbeing needs to be considered when trying to discover why a person may be at a higher risk of burnout.^43^ Personal characteristics, such as self‐esteem, optimism and resilience have been shown to increase work engagement and therefore reduce the risk of burnout. This can also be true for authentic leadership, and other organisational resources that can reduce job demands and stimulate personal growth.^43^

## Limitations

Whilst this study had a small response rate (12.5%) and therefore is not representative of the population of NMTs working in Australia and New Zealand, it is still a reasonable sample of the population and gives an indication as to the burnout levels of NMTs working in Australia and New Zealand. There has been a decline in response rates in health professional surveys over the years,^44^, ^45^ even though online surveys are an effective method of obtaining data from a large population group.^46^ This decline could be attributed to survey fatigue, as well as an overload of surveys being sent to people looking for opinions and feedback on many different topics. The avenues for promotion of the survey were also limited for the research team, and consequently the survey may not have reached all registered NMTs in Australia and New Zealand.

Another limitation of this study is that it may have an element of responder bias and sampling error,^47^, ^48^ as there was a large number of registered NMTs that did not complete the questionnaire. The sample may not be indicative of the whole NMT population as the potential responses from non‐responders may have differed from those who completed the questionnaire.^48^ Multiple reminders to complete the questionnaire were sent through all recruitment channels to try and mitigate this error.

## Conclusion

The literature exploring occupational burnout in NMTs working in Australia and New Zealand is limited. The results of this study report that the majority of participants reported a moderate level of occupational burnout and there were little differences between selected participant subgroups. This level of occupational burnout could have far‐reaching implications on the nuclear medicine community, including decreased patient safety and increased turnover of staff, as well as added health risks to the participants. The results of this survey raise awareness of the moderate burnout levels for the NMT community in Australia and New Zealand. Moving forward, individuals and organisations should develop strategies to reduce the risk of burnout occurring, and self‐care knowledge and strategies must be included in undergraduate and graduate training of NMTs.

## Conflict of Interest

The authors declare no conflict of interest.

## Ethics Approval

University of Newcastle Human Research Ethics Approval was obtained prior to the commencement of the study (H‐2020‐0085).

## Supporting information


**Appendix S1.** Questionnaire.

## Data Availability

The data that support the findings of this study are available from the corresponding author upon reasonable request.
