# Supplementary material for: Occupational burnout in nuclear medicine technologists working in Australia and New Zealand – results of a multi‐national survey
Source: J Med Radiat Sci. 2024 Oct 27;72(1):25–33. doi: 10.1002/jmrs.834 (PMC11909702; doi:10.1002/jmrs.834)
Supplement: Supplementary file 1 — Appendix S1. Questionnaire. [file JMRS-72-25-s001.docx]

# **PART A: DEMOGRAPHICS**

1. Are you currently a nuclear medicine technologist working in Australia?
   - Yes
   - No (questionnaire finishes for these participants)
2. How do you identify as?

- Male
- Female
- Another gender identity, specify if you wish
- I prefer not to answer

1. What is your age?

- 20-29
  - 30-39
  - 40-49
  - 50-59
  - 60 +

1. What is your marital status?
   - Single
   - Engaged
   - Married
   - De facto Relationship
   - Separated
   - Divorced
   - Widowed
   - Other (please specify) …………………………
2. Do you have any dependent children living at home?
   - Yes
   - No

5a. If YES, How many dependent children living at home do you have?

- - - 1
    - 2
    - 3
    - 4
    - 5
    - More than 5

1. Do you have any carer commitments (a carer is anyone who cares, unpaid, for a friend or family member who due to age, illness, disability, a mental health problem or an addiction, cannot cope without their support)?
   - Yes
   - No

6a. If YES, Who do you provide care for? (Tick all that apply)

- - - - Child/ren
      - Parents
      - Grandparents
      - Spouse/Partner
      - Friend
      - Other family member
      - Other (please specify) ……………………

1. How many years have you been a practicing Nuclear Medicine Technologist (NMT)?
   - 0-5 years
   - 6-10 years
   - 11-15 years
   - 16-20 years
   - 21-25 years
   - 26-30 years
   - 30 + years
2. Which country have you practiced in as a NMT (for the majority of your career)?
   - Australia
   - New Zealand
   - 8a. If you have practiced in Australia for the majority of your career, what state/territory are you currently employed in?
   - NSW
   - QLD
   - VIC
   - SA
   - WA
   - Tasmania
   - ACT
   - NT
3. What area do you work in (according to the Modified Monash Model 2019)?

| Modified Monash Category | Description |
| --- | --- |
| MM1 | Metropolitan areas: Major cities accounting for 70% of Australia’s population |
| MM2 | Regional centres: Inner and Outer Regional areas that are in, or within a 20km drive of a town with over 50,000 residents |
| MM3 | Large rural towns: Inner and Outer regional areas that are in, or within a 15km drive of a town with between 15,000 to 50,000 residents |
| MM4 | Medium rural towns: Inner and Outer regional areas that are in, or within a 10 km drive of a town with between 5,000 and 15,000 residents (that are not MM2 or MM3) |
| MM5 | Small rural towns: all remaining inner and outer regional areas |
| MM6 | Remote communities: remote mainland areas and remote islands less than 5km offshore |
| MM7 | Very remote communities: very remote areas and all other remote islands more than 5km offshore |

- - Metropolitan Area (MM1)
  - Regional Centre (MM2)
  - Large Rural Town (MM3)
  - Medium Rural Town (MM4)
  - Small Rural Town (MM5)
  - Remote Community (MM6)
  - Very Remote Community (MM7)
  - New Zealand

1. What type of imaging department do you work in?
   - Public Hospital
   - Private Hospital
   - Private Practice within a Private Hospital
   - Private Practice
   - Other (please specify) …………………………….
2. What type of service(s) does your department offer?
   - General Nuclear Medicine (NM) only
   - General NM and BMD
   - PET/CT only
   - General NM and PET/CT
   - General NM, PET/CT and BMD
   - General NM, PET/CT and radionuclide therapy
   - General NM, PET/CT, BMD and radionuclide therapy
   - PET/CT and radionuclide therapy
   - Other
3. How are you employed?
   - Full-time
   - Part-time
   - Casual
   - Other
4. On average, how many hours per week do you work?
   - 0-10 hrs
   - 11-20 hours
   - 21-30 hours
   - 30-35 hours
   - 35 + hours
5. Are you rostered to do on-call on a regular basis?
   - Yes
   - No

14a. If YES, How often are you rostered to do on-call?

- - - Weekly
    - Fortnightly
    - Monthly
    - Quarterly

14b. When you are on-call, on average how often do you get called in?

- - - Daily
    - 3-5 times per week
    - 1-2 times per week
    - Rarely get called in

1. What is your main role within your department?
   - Chief Technologist
   - Deputy Chief Technologist
   - Senior Technologist
   - Technologist
   - Research Technologist
   - Other (please specify) ……………………..
2. How many technologists are employed in your department (include full-time/part-time/casual technologists)?

| Type of Employment | Number of Technologists |
| --- | --- |
| Full-Time |  |
| Part-Time |  |
| Casual |  |

1. On average, how many patients are scanned in your department per day?
   - Less than 6
   - 6-10
   - 11-20
   - 21-30
   - 31-40
   - 40+

# **PART B: ASSESSMENT OF BURNOUT**

1. The definition of stress is “A state of mental or emotional strain or tension resulting from adverse or demanding circumstances”.

On a scale of 1 (no stress) to 10 (extremely stressed), how stressed are you because of **your working environment**?

**Today:**

| Not stressed at all |  |  |  | Moderately stressed |  |  |  |  | Extremely stressed |
| --- | --- | --- | --- | --- | --- | --- | --- | --- | --- |
| 1 | 2 | 3 | 4 | 5 | 6 | 7 | 8 | 9 | 10 |

**In the past 7 days:**

| Not stressed at all |  |  |  | Moderately stressed |  |  |  |  | Extremely stressed |
| --- | --- | --- | --- | --- | --- | --- | --- | --- | --- |
| 1 | 2 | 3 | 4 | 5 | 6 | 7 | 8 | 9 | 10 |

**In the past month:**

| Not stressed at all |  |  |  | Moderately stressed |  |  |  |  | Extremely stressed |
| --- | --- | --- | --- | --- | --- | --- | --- | --- | --- |
| 1 | 2 | 3 | 4 | 5 | 6 | 7 | 8 | 9 | 10 |

**In the past year:**

| Not stressed at all |  |  |  | Moderately stressed |  |  |  |  | Extremely stressed |
| --- | --- | --- | --- | --- | --- | --- | --- | --- | --- |
| 1 | 2 | 3 | 4 | 5 | 6 | 7 | 8 | 9 | 10 |

1. On a scale of 1 (no stress) to 10 (extremely stressed), how stressed are you **outside of your working environment**?

**Now:**

| Not stressed at all |  |  |  | Moderately stressed |  |  |  |  | Extremely stressed |
| --- | --- | --- | --- | --- | --- | --- | --- | --- | --- |
| 1 | 2 | 3 | 4 | 5 | 6 | 7 | 8 | 9 | 10 |

**In the past 7 days:**

| Not stressed at all |  |  |  | Moderately stressed |  |  |  |  | Extremely stressed |
| --- | --- | --- | --- | --- | --- | --- | --- | --- | --- |
| 1 | 2 | 3 | 4 | 5 | 6 | 7 | 8 | 9 | 10 |

**In the last month:**

| Not stressed at all |  |  |  | Moderately stressed |  |  |  |  | Extremely stressed |
| --- | --- | --- | --- | --- | --- | --- | --- | --- | --- |
| 1 | 2 | 3 | 4 | 5 | 6 | 7 | 8 | 9 | 10 |

**In the last year:**

| Not stressed at all |  |  |  | Moderately stressed |  |  |  |  | Extremely stressed |
| --- | --- | --- | --- | --- | --- | --- | --- | --- | --- |
| 1 | 2 | 3 | 4 | 5 | 6 | 7 | 8 | 9 | 10 |

**PROFESSIONAL QUALITY OF LIFE SCALE (ProQOL)**

When you help people, you have direct contact with their lives. As you may have found, your compassion for those you help can affect you in positive and negative ways. Below are some questions about your experiences, both positive and negative, as a nuclear medicine technologist.

Consider each of the following questions about you and your ***current work situation***. Select the number that honestly reflects how frequently you experienced these things in ***the last 30 days***.

| Question | Never | Rarely | Sometimes | Often | Very Often |
| --- | --- | --- | --- | --- | --- |
| 1. I am happy. | 1 | 2 | 3 | 4 | 5 |
| 2. I am preoccupied with more than one person I have scanned. | 1 | 2 | 3 | 4 | 5 |
| 3. I get satisfaction from being able to help people. | 1 | 2 | 3 | 4 | 5 |
| 4. I feel connected to others. | 1 | 2 | 3 | 4 | 5 |
| 5. I jump or am startled by unexpected sounds. | 1 | 2 | 3 | 4 | 5 |
| 6. I feel invigorated after working with those I help. | 1 | 2 | 3 | 4 | 5 |
| 7. I find it difficult to separate my personal life from my life as a nuclear medicine technologist. | 1 | 2 | 3 | 4 | 5 |
| 8. I am not as productive at work because I am losing sleep over traumatic experiences of a person I have scanned. | 1 | 2 | 3 | 4 | 5 |
| 9. I think that I might have been affected by the traumatic stress of those I have scanned. | 1 | 2 | 3 | 4 | 5 |
| 10. I feel trapped by my job as a nuclear medicine technologist. | 1 | 2 | 3 | 4 | 5 |
| 11. Because of my role or career, I have felt "on edge" about various things. | 1 | 2 | 3 | 4 | 5 |
| 12. I like my work as a nuclear medicine technologist. | 1 | 2 | 3 | 4 | 5 |
| 13. I feel depressed because of the traumatic experiences of the people I have scanned. | 1 | 2 | 3 | 4 | 5 |
| 14. I feel as though I am experiencing the trauma of someone I have scanned. | 1 | 2 | 3 | 4 | 5 |
| 15. I have beliefs that sustain me. | 1 | 2 | 3 | 4 | 5 |
| 16. I am pleased with how I am able to keep up with nuclear medicine techniques and protocols. | 1 | 2 | 3 | 4 | 5 |
| 17. I am the person I always wanted to be. | 1 | 2 | 3 | 4 | 5 |
| 18. My work makes me feel satisfied. | 1 | 2 | 3 | 4 | 5 |
| 19. I feel worn out because of my work as a nuclear medicine technologist | 1 | 2 | 3 | 4 | 5 |
| 20. I have happy thoughts and feelings about those I scan and how I could help them. | 1 | 2 | 3 | 4 | 5 |
| 21. I feel overwhelmed because my workload seems endless. | 1 | 2 | 3 | 4 | 5 |
| 22. I believe I can make a difference through my work. | 1 | 2 | 3 | 4 | 5 |
| 23. I avoid certain activities or situations because they remind me of frightening experiences of the people I have scanned. | 1 | 2 | 3 | 4 | 5 |
| 24. I am proud of what I can do to as a nuclear medicine technologist. | 1 | 2 | 3 | 4 | 5 |
| 25. As a result of my career, I have intrusive, frightening thoughts. | 1 | 2 | 3 | 4 | 5 |
| 26. I feel "bogged down" by the system. | 1 | 2 | 3 | 4 | 5 |
| 27. I have thoughts that I am a "success" as a nuclear medicine technologist. | 1 | 2 | 3 | 4 | 5 |
| 28. I can't recall important parts of my work with trauma victims. | 1 | 2 | 3 | 4 | 5 |
| 29. I am a very caring person. | 1 | 2 | 3 | 4 | 5 |
| 30. I am happy that I chose to do this work. | 1 | 2 | 3 | 4 | 5 |

# **PART C: FACTORS CONTRIBUTING TO OCCUPATIONAL BURNOUT**

1. Is it easy or difficult to organise leave at your workplace?

Sick Leave:

- - - Easy
    - Somewhat easy
    - Neutral
    - Somewhat difficult
    - Very difficult

Personal Leave:

- - - Easy
    - Somewhat easy
    - Neutral
    - Somewhat difficult
    - Very difficult

Annual Leave:

- - - Easy
    - Somewhat easy
    - Neutral
    - Somewhat difficult
    - Very difficult

Long Service Leave (if eligible):

- - - Easy
    - Somewhat easy
    - Neutral
    - Somewhat difficult
    - Very difficult

Comments: …………………………….

1. When was the last time you had leave?

Sick Leave:

- Within the last month
- Within the last 2-3 months
- Within the last 4-6 months
- Within the last 7-9 months
- Within the last 10-12 months
- Longer than 12 months ago

Personal Leave:

- Within the last month
- Within the last 2-3 months
- Within the last 4-6 months
- Within the last 7-9 months
- Within the last 10-12 months
- Longer than 12 months ago

Annual Leave:

- Within the last month
- Within the last 2-3 months
- Within the last 4-6 months
- Within the last 7-9 months
- Within the last 9-12 months
- Longer than 12 months ago

Long Service Leave (if eligible):

- Within the last month
- Within the last 2-3 months
- Within the last 4-6 months
- Within the last 7-9 months
- Within the last 10-12 months
- Longer than 12 months ago

Comments: ……………………………………………………………..

1. Indicate how the following situations make you feel.

| Situation | Never stressed | Rarely stressed | Sometimes stressed | Often stressed | Always stressed |
| --- | --- | --- | --- | --- | --- |
| Scanning paediatric patients | 1 | 2 | 3 | 4 | 5 |
| Scanning elderly patients | 1 | 2 | 3 | 4 | 5 |
| Scanning very ill patients | 1 | 2 | 3 | 4 | 5 |
| Low staffing levels | 1 | 2 | 3 | 4 | 5 |
| Low pay rate | 1 | 2 | 3 | 4 | 5 |
| Busy workload | 1 | 2 | 3 | 4 | 5 |
| Quiet workload | 1 | 2 | 3 | 4 | 5 |
| Too many working hours | 1 | 2 | 3 | 4 | 5 |
| Too little working hours | 1 | 2 | 3 | 4 | 5 |
| Having to do overtime regularly | 1 | 2 | 3 | 4 | 5 |
| Having to be on the on-call roster | 1 | 2 | 3 | 4 | 5 |
| Getting called in during the week/on the weekend | 1 | 2 | 3 | 4 | 5 |
| Coming in on a day off for a meeting | 1 | 2 | 3 | 4 | 5 |
| Coming in on a day off for training | 1 | 2 | 3 | 4 | 5 |
| Coming in early/staying late for a meeting | 1 | 2 | 3 | 4 | 5 |
| Coming in early/staying late for training | 1 | 2 | 3 | 4 | 5 |
| Lack of career progression opportunities | 1 | 2 | 3 | 4 | 5 |
| Lack of recognition of your work by department management | 1 | 2 | 3 | 4 | 5 |
| The type of work you perform - hotlab duties | 1 | 2 | 3 | 4 | 5 |
| The type of work you perform - scanning patients | 1 | 2 | 3 | 4 | 5 |
| The type of work you perform – administering therapy to patients | 1 | 2 | 3 | 4 | 5 |
| The type of work you perform - administrative duties | 1 | 2 | 3 | 4 | 5 |
| Continuing professional development tasks | 1 | 2 | 3 | 4 | 5 |
| Relationships between work colleagues and yourself | 1 | 2 | 3 | 4 | 5 |
| The way you are treated by patients | 1 | 2 | 3 | 4 | 5 |
|  |  |  |  |  |  |

1. How often do you take your work stresses home with you?

| Never | Once in a while | About half the time | Most of the time | Always |
| --- | --- | --- | --- | --- |
| 0% | 25% | 50% | 75% | 100% |

1. Indicate which of the following events have occurred to you within the past 12 months. How have they made you feel? (On a scale of 1-5, with 1 being not stressed and 5 being extremely stressed)

| Event | N/A | Not stressed | A little stressed | Moderately stressed | Stressed | Extremely stressed |
| --- | --- | --- | --- | --- | --- | --- |
| Family members beginning/ending school | 0 | 1 | 2 | 3 | 4 | 5 |
| Family members begin/end working life | 0 | 1 | 2 | 3 | 4 | 5 |
| Change in residence | 0 | 1 | 2 | 3 | 4 | 5 |
| Financial hardship | 0 | 1 | 2 | 3 | 4 | 5 |
| Death of a close family member/friend | 0 | 1 | 2 | 3 | 4 | 5 |
| Death of a spouse/child | 0 | 1 | 2 | 3 | 4 | 5 |
| Divorce/separation | 0 | 1 | 2 | 3 | 4 | 5 |
| Personal injury/illness | 0 | 1 | 2 | 3 | 4 | 5 |
| Serious injury/illness of a family member/friend | 0 | 1 | 2 | 3 | 4 | 5 |
| Getting married | 0 | 1 | 2 | 3 | 4 | 5 |

# **PART D: METHODS USED TO ALLEVIATE STRESS**

1. Which of these methods do you use to alleviate stress in the workplace?

- Confide in co-worker/s
- Inform your manager
- Write about it in a journal
- Take regular breaks throughout the day
- Talk to friends outside of work in breaks
- Take regular annual/personal leave
- Confide in an independent person within the workplace
- Inform upper management
- Go for a walk outside during your lunch break
- Seek professional help outside of the workplace
- Delegate some of your tasks to others
- Snack on junk food
- Drink alcohol after work hours
- Take illicit drugs after work hours
- Other ……………………………….

1. Does your workplace have any interventions in place to help manage/alleviate stress?
   - Yes
   - No
   - Unsure, have not been informed

26a. If YES, what are these interventions?

- - - - - Buddy/Mentor system
        - Counselling service
        - Meditation/Relaxation classes
        - More flexible work hours
        - Exercise classes/Walking groups
        - Debrief meetings on a regular basis
        - Other………………………………

26b. Have you ever used an intervention provided by your workplace?

- - - Yes
    - No

25b.1 If YES, which intervention(s) did you participate in?

Please specify ……………………………………….

25b.2 Was it effective in reducing your stress levels?

- - - - - Yes
        - No

Comment: ……………………………………………………..

25b.2a Why was it effective in reducing your stress levels at work?

Comment: …………………………………………………….

25.b.2b Why was it not effective in reducing your stress levels at work?

Comment: ……………………………………………………

1. Are there any interventions that you would like to see be implemented by your workplace to help manage/alleviate stress?

……………………………………………………………………………………………………

1. How often do you participate in the following activities outside of work hours to assist with your mental wellbeing?

| Activity | Never | A few times a year | Once a Month | A few times a month | Once a week | A few times a week | Daily |
| --- | --- | --- | --- | --- | --- | --- | --- |
| Cycle | 0 | 1 | 2 | 3 | 4 | 5 | 6 |
| Walk | 0 | 1 | 2 | 3 | 4 | 5 | 6 |
| Run | 0 | 1 | 2 | 3 | 4 | 5 | 6 |
| Swim | 0 | 1 | 2 | 3 | 4 | 5 | 6 |
| Group exercise class | 0 | 1 | 2 | 3 | 4 | 5 | 6 |
| Gym workout | 0 | 1 | 2 | 3 | 4 | 5 | 6 |
| Art (draw, paint etc) | 0 | 1 | 2 | 3 | 4 | 5 | 6 |
| Photography | 0 | 1 | 2 | 3 | 4 | 5 | 6 |
| Get a massage | 0 | 1 | 2 | 3 | 4 | 5 | 6 |
| Yoga/Pilates | 0 | 1 | 2 | 3 | 4 | 5 | 6 |
| Gardening | 0 | 1 | 2 | 3 | 4 | 5 | 6 |
| Meditation/relaxation exercises | 0 | 1 | 2 | 3 | 4 | 5 | 6 |
| Have a relaxing bath | 0 | 1 | 2 | 3 | 4 | 5 | 6 |
| Listen to music | 0 | 1 | 2 | 3 | 4 | 5 | 6 |
| Cook | 0 | 1 | 2 | 3 | 4 | 5 | 6 |
| Crosswords/sudoku/word games (on paper or electronically) | 0 | 1 | 2 | 3 | 4 | 5 | 6 |
| Eat healthily | 0 | 1 | 2 | 3 | 4 | 5 | 6 |
| Drink alcohol | 0 | 1 | 2 | 3 | 4 | 5 | 6 |
| Smoke cigarettes/vape | 0 | 1 | 2 | 3 | 4 | 5 | 6 |
| Take illicit drugs | 0 | 1 | 2 | 3 | 4 | 5 | 6 |
| Snack on junk food | 0 | 1 | 2 | 3 | 4 | 5 | 6 |
| Counselling sessions | 0 | 1 | 2 | 3 | 4 | 5 | 6 |
| Spend time with friends | 0 | 1 | 2 | 3 | 4 | 5 | 6 |
| Spend time with family | 0 | 1 | 2 | 3 | 4 | 5 | 6 |
| Go shopping | 0 | 1 | 2 | 3 | 4 | 5 | 6 |
| Go to the beach | 0 | 1 | 2 | 3 | 4 | 5 | 6 |
| Read | 0 | 1 | 2 | 3 | 4 | 5 | 6 |
| Play computer games (including Xbox etc) | 0 | 1 | 2 | 3 | 4 | 5 | 6 |
| Go for a drive/motorbike ride | 0 | 1 | 2 | 3 | 4 | 5 | 6 |
| Watch a movie/TV | 0 | 1 | 2 | 3 | 4 | 5 | 6 |

**Additional comments**: Do you have any other comments you would like to add with regards to workplace stress and occupational burnout amongst nuclear medicine technologists working in Australia and New Zealand?

…………………………………………………………………………………………………………………………………………………………………………………………………………………………………………………………………………………………………………………………………………………………………………………………………………………………………………………………………………………………………………………………………………………………………………………………………………………………………………………………………………………………………………………………………………………………………………………………………………………………………………………………………………………………………………

**Thank you for your time and input. The information you have provided will help us assess burnout in nuclear medicine technologists working in Australia and New Zealand**.
